# Cell-Free Protein Expression by a Reconstituted Transcription–Translation System Energized by Sugar Catabolism

**DOI:** 10.3390/molecules29132956

**Published:** 2024-06-21

**Authors:** Gaku Sato, Shintaro Miyazawa, Nobuhide Doi, Kei Fujiwara

**Affiliations:** Department of Biosciences & Informatics, Keio University, 3-14-1 Hiyoshi, Kohoku-ku, Yokohama 223-8522, Japan

**Keywords:** glycolysis, cell-free protein synthesis, bottom-up synthetic biology

## Abstract

Cooperation between catabolism and anabolism is crucial for maintaining homeostasis in living cells. The most fundamental systems for catabolism and anabolism are the glycolysis of sugars and the transcription–translation (TX-TL) of DNA, respectively. Despite their importance in living cells, the in vitro reconstitution of their cooperation through purified factors has not been achieved, which hinders the elucidation of the design principle in living cells. Here, we reconstituted glycolysis using sugars and integrated it with the PURE system, a commercial in vitro TX-TL kit composed of purified factors. By optimizing key parameters, such as glucokinase and initial phosphate concentrations, we determined suitable conditions for their cooperation. The optimized system showed protein synthesis at up to 33% of that of the original PURE system. We observed that ATP consumption in upstream glycolysis inhibits TX-TL and that this inhibition can be alleviated by the co-addition of glycolytic intermediates, such as glyceraldehyde 3-phosphate, with glucose. Moreover, the system developed here simultaneously synthesizes a subset of its own enzymes, that is, glycolytic enzymes, in a single test tube, which is a necessary step toward self-replication. As glycolysis and TX-TL provide building blocks for constructing cells, the integrated system can be a fundamental material for reconstituting living cells from purified factors.

## 1. Introduction

Living cells obtain energy and small metabolites by catabolizing nutrients and anabolizing them into large biomolecules. The most fundamental catabolic pathway is glycolysis, which converts sugars like glucose (Glc) into pyruvate. The molecules synthesized by glycolysis, including their intermediates, are further converted into carbon building blocks such as nucleic acids, amino acids, and phospholipids [1,2,3]. In addition to its role as a carbon building block supplier, glycolysis synthesizes energy such as ATP for biochemical systems [1,4]. 

Among sugars, glucose is the most preferred for glycolysis, as it is the sole carbon source of many organisms in the three domains of life [2,5,6] and is consumed in preference to other sugars to achieve fast growth [7]. The energy obtained from glucose is used for anabolism, which converts small molecules into macromolecules. The most fundamental anabolic mechanism is the transcription–translation (TX-TL) of genes. Cooperation between catabolism and anabolism is the essence of the self-replication of living cells. Therefore, the reconstitution of this cooperation is necessary to elucidate the design principles of living cells and can contribute to the in vitro creation of living systems.

Anaerobic glycolysis, or fermentation, is a branch of glycolysis that converts pyruvate, the final product of glycolysis, into lactate or ethanol for NADH oxidation. Anaerobic glycolysis yields a total of two ATPs from a single glucose molecule. Although the ATP yield is approximately 10 times lower than that of respiration, it is sufficient to cover all intracellular systems for the self-replication of living cells. Anaerobic glycolysis is a powerful energy regeneration system in extract-based cell-free protein expression systems that produce more than 1 mg/mL of protein [8,9,10,11]. This high protein yield is the result of proper cooperation between glycolysis and TX-TL. However, because the components of the extract are unclear, reconstitution of the integrated system of glycolysis and TX-TL from purified factors is necessary to understand their cooperativity. 

To this end, we modified the PURE system [12], an *E. coli* TX-TL system reconstituted with purified factors, and replaced it with a creatine–phosphate and creatine kinase (CP-CK) energy regeneration system with glycolysis [13]. We observed metabolic competition between glycolysis and the TX-TL system for successful cooperation and succeeded in reconstituting the integration of glycolysis and TX-TL using purified factors in the case of glucose 6-phosphate (G6P) as the initial substrate (G-PURE). G6P is the initial substrate for bacterial glycolysis [14]. However, many organisms uptake sugars, such as glucose, as the initial substrate [2,15,16,17]. The next step in the in vitro creation of living systems is to confer sugar catabolism to G-PURE. 

G6P is the product of the first glycolytic enzyme, glucokinase (Glk), which phosphorylates glucose using ATP. To confer G-PURE with glucose catabolism ability, Glk must be added to G-PURE. However, the addition of Glk to G-PURE drastically changes its dynamics. Our previous study showed that the competition for ATP between glycolysis and TX-TL regulates G-PURE activity [13]. Because of the competition for ATP, the initial concentration of common components, especially inorganic phosphate (P_i_), determines the fate of the efficient coupling of the integrated system. Glk complicates the competition because it consumes ATP. Glucose-start glycolysis consumes ATP twice in the upper part (Glk and PfkA), called “turbo design” [18]. Many studies, including mathematical modeling and in vivo and in vitro experiments, have shown that this turbo design causes fatal metabolic collapse unless Glk is strictly regulated [18,19,20,21,22,23,24,25]. Briefly, strong Glk activity depletes the ATP available for the second ATP-consuming reaction of glycolysis (PfkA), whereas weak Glk activity slows glycolysis. Living cells deal with the turbo design through feedback regulation to realize glucose catabolism [20]. This should be considered when reconstituting the integration of sugar-based glycolysis and the TX-TL system.

Herein, we report the successful reconstitution of a cell-free protein expression system energized by glucose using purified factors (SG-PURE). We observed that glyceraldehyde 3-phosphate (GAP), an intermediate of glycolysis, helped smooth system integration. Although SG-PURE exhibited a lower protein yield than that of the commercial CP-CK-based PURE system, it was able to synthesize various proteins, including glycolytic enzymes. The achievements of this study pave the way for the creation of synthetic cells that catabolize glucose and anabolize proteins and provide a tool for the construction of in vitro systems with self-replication ability. 

## 2. Results

### 2.1. In Vitro Reconstitution of Glycolysis Using Glucose as an Initial Substrate

**Figure 1:**
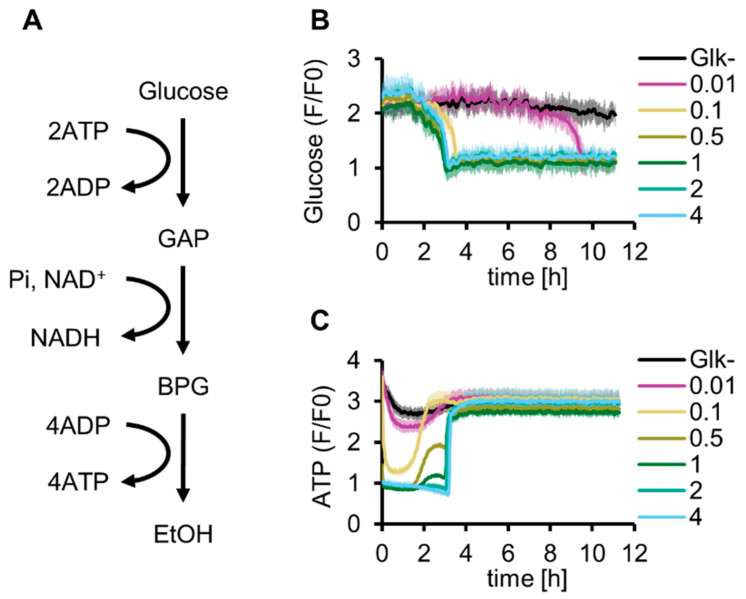
Optimization of Glk concentration for in vitro reconstitution of glycolysis. (**A**) Schematics of glycolysis. (**B**) Glucose dynamics of glycolysis under various Glk concentrations (µM) at 30 °C. (**C**) ATP dynamics of glycolysis under various Glk concentrations (µM) at 30 °C. (**B**,**C**) These data are measured simultaneously in the same samples. All measurements were *n* = 3. Means and standard deviations are shown.

First, we reconstituted glucose-start glycolysis by adding purified Glk to the reconstituted G6P-start glycolysis developed in our previous study [13]. As explained by the turbo design model [18], the concentration of Glk is a critical variable for glucose-start glycolysis because abundant Glk depletes the ATP available for other reactions, whereas a shortage of Glk limits glycolytic activity (Figure 1A and Figure S1A). To investigate the optimal Glk concentration for the reconstituted glycolysis, glucose consumption and ATP synthesis were simultaneously tracked using Green Glifon4000 (a fluorescent glucose sensor [26]) and MaLionR (a fluorescent ATP sensor [27]) (Figure 1B,C). The time profiles of fluorescence intensities indicated that glucose concentrations gradually decreased over time and were below the detection limit (1 mM) in approximately 3 h under 0.1 µM or higher Glk conditions (Figure S2A). With 0.01 µM Glk, the glucose concentration decreased to reach the same level after 10 h. In contrast to the simple consumption of glucose during the reaction, the dynamics of the ATP concentration showed a more complex behavior. Immediately after the initiation of the reaction, a low ATP concentration was maintained for several hours. After this low-concentration period, the ATP concentrations increased rapidly and plateaued at the initial ATP concentration. The lowest ATP concentration at the initial stage depended on Glk concentration, and the ATP levels became lower than the detection limit of MaLionR under higher Glk conditions (0.5 µM or more, Figure S2B). Further, the time at which the ATP concentrations increased became longer at higher Glk concentrations. The recovery of ATP concentrations under 0.5 and 1 µM Glk conditions exhibited a two-step profile. Recovery stopped once at approximately 2 h and then increased again to a level similar to the initial ATP concentration. The ATP concentration did not exceed the initial ATP concentration through energy regeneration using glycolysis because ADP was not initially added in this experiment. The complex dynamics of the ATP concentration suggest that the Glk concentration is a critical factor for managing glycolytic flux.

### 2.2. Construction of G-PURE Energized by Sugar

**Figure 2:**
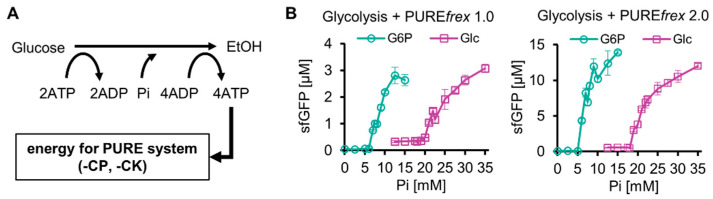
Construction of SG-PURE. (**A**) Schematics of SG-PURE. (**B**) P_i_ dependence of G-PURE and SG-PURE. Note that this experiment was performed under the conditions of 15 mM Mg, 25 mM glucose, and 1 µM Glk. The data on cell-free protein expression using G6P as a substrate were obtained in our previous study [13]. All measurements were *n* = 3, except 12.5 mM P_i_ (*n* = 6). Means and standard deviations are shown.

Next, the reconstituted glucose-start glycolysis was integrated with a reconstituted TX-TL system by replacing the ATP regeneration system (creatine phosphate/kinase system, CP-CK system) of PURE*frex* 1.0 or PURE*frex* 2.0 with glucose-start glycolysis. PURE*frex* 1.0 and PURE*frex* 2.0 are versions of the commercial PURE system, and PURE*frex* 2.0 shows approximately 10-fold higher protein synthesis than that with PURE*frex* 1.0. There are two types of commercial PURE systems: PURExpress (NEB) and PURE*frex* (GeneFrontier). They differ in the absence or presence of His-tag on the constituent proteins and other components. Because of its higher protein yields, we used PURE*frex* in this study. Because the resultant integrated system was expected to function as a cell-free protein expression system energized by sugars such as glucose (Figure 2A), we termed the system a purified protein expression system using sugar-based glycolysis as an energy source (SG-PURE). Depending on the version of the PURE system, we termed the systems SG-PURE 1.0 and SG-PURE 2.0, based on PURE*frex*1.0 and 2.0, respectively. 

Our previous study with G-PURE, which used G6P-start glycolysis as an energy source, revealed that the most critical factor for protein expression using G6P-start glycolysis was the initial P_i_ concentration [13]. This was the case for SG-PURE, though SG-PURE required two-fold higher P_i_ supplementation for efficient protein expression than G-PURE (Figure 2B). SG-PURE required approximately the same amount of P_i_ as glucose, whereas G-PURE required approximately half the amount of P_i_ as G6P. These differences in the thresholds of initial P_i_ concentrations for efficient protein expression between the glucose- and G6P-start PURE systems did not depend on the version of the PURE system. P_i_ was less required in the case of SG-PURE 2.0 than in SG-PURE 1.0, corresponding to the previous G-PURE using G6P as the substrate. 

The reason why SG-PURE requires much more P_i_ than G-PURE is as follows: Our previous study showed that the kinetic balance between glycolysis and protein synthesis, rather than the net balance of reactions, is important for their coupling [13]. This is because upstream glycolysis and protein synthesis compete for ATP, and upstream glycolysis does not produce P_i_. Due to the competition, P_i_ from the ATPase reaction induced by protein synthesis is necessary for coupling. If the P_i_ levels are insufficient, ATP regeneration from ADP created by downstream glycolysis does not proceed. This is the reason why the initial addition of P_i_ is necessary. Because glycolysis consumes two lots of ATP per one lot of glucose and one lot of ATP per one lot of G6P, much P_i_ is required for glucose-start glycolysis. Consequently, SG-PURE was supposed to require two-fold higher Pi supplementation than G-PURE.

### 2.3. Optimization of Cell-Free Protein Expression by SG-PURE

**Figure 3:**
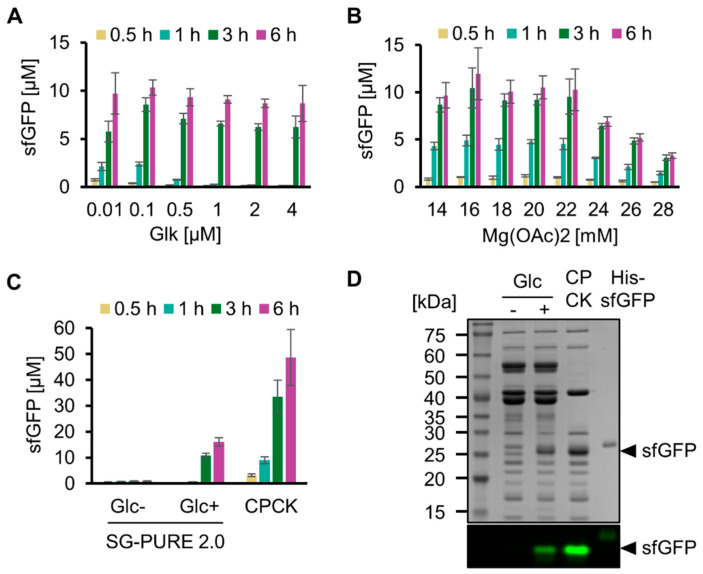
Optimization of SG-PURE. (**A**) Optimization of Glk concentration and (**B**) Mg concentration for sfGFP expression with SG-PURE. (**C**) Comparison of SG-PURE with commercial PUREfrex 2.0, which uses CP-CK as an energy source. (**D**) SDS-PAGE of the cell-free protein expression proteins. Proteins were detected by CBB staining after boiling and by sfGFP fluorescence in non-boiled samples, detected using a fluorescence imager. (**A**–**C**) Means and standard deviations are shown (*n* = 3).

We then optimized the concentrations of key components of SG-PURE to improve protein yield. Hereafter, we used SG-PURE 2.0 because it showed a 5-fold higher protein yield than that of SG-PURE 1.0. For protein synthesis using SG-PURE 2.0, P_i_ was added at the same concentration as that of glucose. First, the Glk concentration was optimized by testing from 0.01 µM to 4 µM (Figure 3A). Consistent with the ATP concentration dynamics of glycolysis with various concentrations of Glk, protein synthesis levels were low at an earlier stage of the reaction at higher Glk concentrations. This result suggests an inhibitory effect of decreased ATP concentration at the beginning of the reaction. From the expression yield at 6 h and the speed of the earlier stage reactions, we concluded that 0.1 µM Glk was optimal for protein synthesis by SG-PURE 2.0. Second, the Mg^2+^ ion concentration was optimized (Figure 3B). The protein yield of the PURE system depends on Mg^2+^, and the optimal Mg^2+^ concentration depends on NTPs and P_i_ concentrations [28]. Because the concentrations of NTPs and P_i_ in SG-PURE 2.0 are different from those in commercial PURE*frex* 2.0, the dependence of protein yields on Mg^2+^ concentration was investigated. Consequently, 16 mM Mg^2+^ was determined as the optimal concentration.

Using SG-PURE 2.0 under optimized conditions, we quantified its protein yield (Figure 3C). SG-PURE 2.0 synthesized 16.0 µM sfGFP (0.43 mg/mL) after a 6 h reaction. Although its level was approximately 33% of that with commercial PURE*frex* 2.0 (1.3 mg/mL), SG-PURE 2.0 exhibited higher protein yields than G-PURE 2.0 (0.3 mg/mL) reported in our previous report [13]. Furthermore, SG-PURE synthesizes more protein than the basic commercial PURE system, which synthesizes less than 0.3 mg/mL of proteins (PUREfrex 1.0 or PURExpress, from the website information). An SDS-PAGE of the expressed protein with or without heat denaturation confirmed the expression of full-length sfGFP (Figure 3D). During the reaction, SG-PURE 2.0 converted 85 ± 5% (*n* = 3) of glucose into ethanol (Figure S4). This indicated that TX-TL was energized by the glycolysis of glucose. 

### 2.4. Effect of Glycolytic Intermediates on Glycolysis and SG-PURE

**Figure 4:**
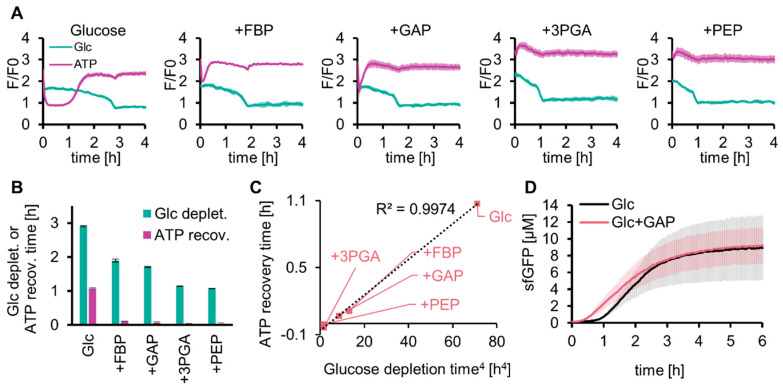
Effect of glycolysis intermediates on glycolysis and SG-PURE. (**A**) Effect of supplementation with glycolysis intermediates on glucose consumption and ATP levels of glycolysis. (**B**) Time for glucose depletion and ATP recovery calculated from (**A**). Means and standard deviations are shown (*n* = 3). (**C**) Correlation between the quadruplicate of the glucose depletion time and the ATP recovery time is shown in (**B**). (**D**) Effect of GAP on SG-PURE 2.0. Glc+GAP and Glc indicate 50 mM glucose with or without 5 mM GAP. Means and standard deviations are shown (*n* = 3).

The long lag time for protein expression with SG-PURE (SG-PURE 2.0) indicated that TX-TL did not work efficiently because of the low level of ATP in the early stages of glycolysis. We investigated whether solving this problem would improve the protein yield of SG-PURE. We hypothesized that the shortage of glycolysis intermediates at an earlier stage of the reaction was the cause of the decrease in ATP concentration and expected that supplementation with the intermediate substrates of glycolysis would solve this problem. To test this hypothesis, 5 mM each of the intermediate substrates of glycolysis (FBP, GAP, 3PGA, and PEP) was co-added with 50 mM glucose, and the glucose and ATP concentrations were tracked over time using Green Glifon4000 and MaLionR (Figure 4A). Supplementation with these intermediates shortened the time required for ATP recovery and glucose consumption (Figure 4B). Intermediates located further downstream of the glycolysis pathway further shortened the lag time. The regression curve suggested that the ATP recovery time was proportional to the quadruplicate of the glucose depletion time (Figure 4C). 

As the addition of glycolytic intermediates shortened the lag time of the ATP concentration increase, we investigated whether their addition to SG-PURE improved protein yield (Figure 4D). We selected GAP as the intermediate for this test. This is because 3PGA and PEP do not use P_i_ and work only as a source for ATP synthesis. The lag time of glycolysis by GAP supplementation was shorter than that with FBP, and the reaction of GAP by GAPDH was located at the separation point of upstream and downstream glycolysis. Supplementation with 5 mM GAP shortened the lag time (from 1 h to 0.5 h). However, GAP supplementation did not improve the final protein yield, indicating that the reason for the lower protein yield with SG-PURE than that with PURE*frex* is neither the lack of energy nor the low ATP concentrations at the earlier stage of the reactions.

### 2.5. Expression of Various Proteins by SG-PURE as a Step toward Self-Replication

**Figure 5:**
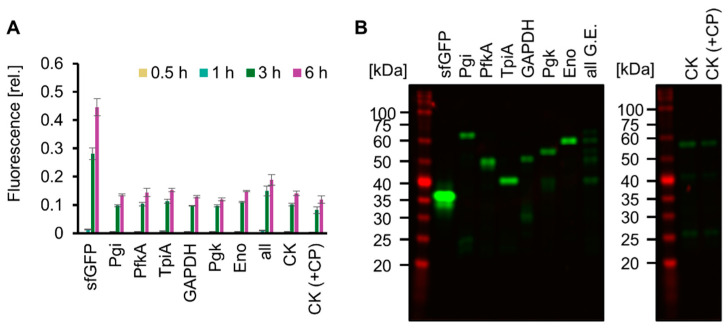
Expression of various proteins by SG-PURE. (**A**) Time course of SG-PURE 2.0 expressing various proteins. Fluorescence intensities were normalized to the fluorescence intensity of sfGFP expressed by PUREfrex 2.0 at 6 h. Note that the range of the *y*-axis is different from that of CPCK (Figure S5A). Means and standard deviations are shown (*n* = 3). (**B**) SDS-PAGE of various proteins expressed by SG-PURE 2.0 without boiling. Note that Pgi, PfkA, TpiA, GAPDH, Pgk, and Eno have the msfGFP tag at their N-terminus. G.E.s, glycolytic enzymes. “All G.E.” indicates the expression of six genes (msfGFP-tagged Pgi, PfkA, TpiA, GAPDH, Pgk, and Eno) in a single tube. CK (+CP) is supplemented with 20 mM CP in addition to 50 mM glucose.

Living cells use ATP obtained from glucose catabolism and express various proteins encoded in the genome using ATP. We mimicked this feature of life by testing whether SG-PURE could synthesize proteins of various lengths, especially glycolytic enzymes, as a step toward self-replication. For detection, each glycolytic enzyme fused with msfGFP at its N-terminus (Pgi, PfkA, TpiA, GAPDH, Pgk, and Eno) was expressed using SG-PURE. Consequently, msfGFP fluorescence increased over time in all the tested genes, indicating the expression of msfGFP-fused glycolytic enzymes (Figure 5A). Full-length expression was confirmed by the molecular weights of the expressed proteins, which were estimated using an SDS-PAGE of non-boiled and heat-denatured samples (Figure 5B and Figure S5). Moreover, SG-PURE was able to synthesize CK, the energy regeneration system of the original PURE system. However, the expression of CK by the co-supplementation of 20 mM CP with glucose did not improve protein yield. This result indicated that SG-PURE already obtained sufficient energy from glucose, and the lower yield than that of the PURE system using CP-CK was not attributed to the energy problem. Finally, we tested the ability of SG-PURE 2.0 for multiple protein synthesis in the same tube. DNA from six glycolytic genes (Pgi, PfkA, TpiA, GAPDH, Pgk, and Eno) was mixed and added to SG-PURE. SDS-PAGE analysis showed that SG-PURE achieved the simultaneous cell-free protein synthesis of all the glycolytic enzymes tested in a single tube (Figure 5B). These results show that SG-PURE can synthesize proteins of various lengths, similar to living cells.

## 3. Discussion

In this study, we reconstituted an integrated system of sugar catabolism (glucose-start glycolysis) and TX-TL using purified factors (SG-PURE). This conclusion is supported by the following results: (i) both glucose and phosphate are necessary for protein expression by SG-PURE (Figure 2 and Figure 3), (ii) an intermediate substrate (GAP) of glycolysis affect the time profile of protein synthesis (Figure 4), and (iii) ethanol, a product of glycolysis, was concomitantly synthesized by the SG-PURE reaction (Figure S4). Efficient protein expression in the integrated system was achieved by supplementation with P_i_ at the same glucose concentrations (Figure 2). After the optimization of the Glk and Mg concentrations, we observed suitable conditions for protein expression using SG-PURE (Figure 3). The SG-PURE developed in this study can express various glycolytic proteins of various sizes, even in a single tube (Figure 5). Because glycolysis has the potential to synthesize the resources for all carbon building blocks of the PURE system and energy from glucose, these achievements are an important step toward realizing the in vitro construction of a self-replication system.

Various studies have reported the replacement of the energy regeneration system of the PURE system with other energy regeneration system, such as polyphosphate kinase, photosynthesis, and Rubisco [29,30,31]. In particular, the polyphosphate kinase-energized PURE systems exhibit a higher protein yield than SG-PURE 2.0. Further improvement in SG-PURE is needed to use SG-PURE as the basis for the construction of artificial cells. However, SG-PURE could be more cost-effective than the CP-CK system, at least. In terms of price, 1 mol of glucose is approximately 700 times less expensive than 1 mol of CP. Moreover, glycolysis can yield two ATPs from one lot of glucose, whereas the CP-CK system obtains one ATP from one CP. Considering the low cost of glucose, further improvement in SG-PURE 2.0 will contribute to reducing the cost of protein expression induced by the PURE system.

The finding that the co-addition of glycolytic intermediates with glucose increased the glycolytic flux deepened our understanding of glycolysis and illuminated the way to eliminate the glycolysis bottleneck. Previous in vitro glycolysis studies have focused on adjusting Glk activity to avoid problems in the turbo design of glycolysis [19,21,22,23,24] because rapid Glk depletes ATP, which inhibits a downstream reaction (PfkA reaction) that uses ATP. However, lowering the Glk concentration weakens the glycolytic flux. We observed that the co-addition of glycolytic intermediates with glucose facilitated glucose consumption (Figure 4). The intermediates used (5 mM) provided only 5 mM (3PGA and PEP), 10 mM (GAP), or 20 mM ATP (FBP), whereas 50 mM glucose required 100 mM ATP in the upstream pathway. In other words, even though the glycolytic intermediates provide 5–20% of the ATP consumed by the upper pathway of glycolysis, they contribute significantly to the maintenance of ATP concentrations and efficient conversion of glucose. These findings elucidate the importance of intermediate substrates for the efficient flux of complex reaction pathways, such as glycolysis, and indicate the importance of flux balance between the upstream and downstream pathways of glycolysis. Moreover, since the ATP profile of extract-based protein synthesis energized by glycolysis is similar to that of SG-PURE [8], it is possible to reduce the lag time of the extract-based system through the supplementation of the glycolysis intermediates.

To date, many studies have expanded the function of the PURE system to realize its self-replication [29,30,31,32,33]. The PURE system has been demonstrated to produce biological processes such as DNA replication and cell division plane determination systems using multiple DNA templates [34,35,36,37,38,39,40,41]. Furthermore, we have previously shown that cell-free protein expression can synthesize proteins using bacterial genomes as a template (in vitro Genome TX-TL, iGeTT) [42], even in liposomes [43], though this ability has not yet been reported in the PURE system. Although the decoding of DNA information into proteins by the PURE system has been well studied, metabolism is the next step toward the reconstitution of living cells from biomolecules. Recently, the PURE system has been expanded to couple with various metabolisms, such as CO_2_ fixation, amino acid synthesis [33,44], and ATP production by Rubisco [31]. These studies showed the synthesis of its own components from nutrients and the applicability of the PURE system to sustainable materials. Although our glycolysis is still limited to the synthesis of energy, glycolysis has the potential to synthesize the resources for all the carbon building blocks of the PURE system other than energy from glucose. Therefore, SG-PURE is an important platform for constructing living cells in vitro. With the addition of findings to overcome the complexity of glycolysis, the SG-PURE system developed here is expected to advance bottom-up synthetic biology.

## 4. Materials and Methods

### 4.1. Materials

Bactotriptone for Green Glifon4000 expression and for others was purchased from Nacalai Tesque (Kyoto, Japan) or BD Bioscience (Franklin Lakes, NJ, USA), respectively. Yeast extract was purchased from BD Bioscience (Franklin Lakes, NJ, USA). Glucose (Glc), fructose 1, 6-bisphosphate (FBP), phosphoenol pyruvate (PEP), Thiamine pyrophosphate (TPP), phenol, 4-aminoantipyrine, NaCl, HEPES, potassium glutamate (GluK), imidazole, magnesium acetate tetrahydrate (Mg(OAc)_2_), ethanol, Tris-HCl pH 7.6, potassium dihydrogen phosphate (KH_2_PO_4_), isopropyl-b-D-thiogalactoside (IPTG), and dipotassium hydrogenphosphate (K_2_HPO_4_) were purchased from Nacalai Tesque (Kyoto, Japan). Nicotinamide adenine dinucleotide (NAD^+^) and adenosine triphosphate disodium salt hydrate (ATP) were purchased from FUJIFILM Wako (Tokyo, Japan). Alcohol oxidase from *Pichia pastoris* and Glycelaldehyde 3-phosphate (GAP) were purchased from Sigma-Aldrich (St. Louis, MO, USA). 3-Phosphoglyceric acid (3PGA) was purchased from Tokyo Chemical Industry Co., Ltd. (Tokyo, Japan). Peroxidase from horseradish was purchased from TOYOBO (Osaka, Japan). Custom PURE*frex* 1.0 and 2.0, in which CP, NTPs, Mg(OAc)_2_, and CK were omitted, were supplied from GeneFrontier (Chiba, Japan). Genes of glycolytic enzymes in the *E. coli* MG1655 genome and a synthetic gene of a mutant of PDC (5SL30, [45]) were cloned in a pHis-SUMO vector. As the template of DNA for the SG-PURE reaction, glycolytic genes were fused with SUMO-msfGFP. These plasmids were constructed in our previous studies [46]. Yeast ADH was purchased from Oriental Yeast (Tokyo, Japan).

The abbreviations for the glycolytic enzymes are as follows: Glk: glucokinase; Pgi: glucose-6-phosphate isomerase; PfkA: 6-phosphofructokinase 1; FbaA: fructose-bisphosphate aldolase class II; TpiA: triose-phosphate isomerase; GAPDH: glyceraldehyde-3-phosphate dehydrogenase; Pgk: phosphoglycerate kinase; GpmA: 2;3-bisphosphoglycerate-dependent phosphoglycerate mutase; Eno: enolase; PykF: pyruvate kinase 1; PykA: pyruvate kinase 2; PDC: pyruvate decarboxylase; and ADH: alcohol dehydrogenase.

### 4.2. Expression and Purification of Glycolytic Enzymes and PDC

*E. coli* BL21 (DE3) RIPL cells (Agilent Technologies, Santa Clara, CA, USA) harboring the plasmid encoding each glycolytic enzyme with a His-SUMO tag on its N-term were cultivated at 37 °C in LB medium with 0.1 mg/mL ampicillin till OD_600_ reached 0.3, and 0.1 mM IPTG was added to induce protein expression. After further cultivation for 5 h at 30 °C, cells were collected by centrifugation (4 °C, 13,000× *g*, 2 min) and resuspended in 10 mL of Lysis buffer (50 mM Tris-HCl pH 7.6, 500 mM NaCl, 20 mM imidazole) per 1 g of wet cell pellet. The cells were sonicated with a tip sonicator (BRANSON, Danbury, CT, USA, Sonifier 250) at power 3 and duty 30% for 30 min on ice. Expressed proteins in the supernatant after centrifugation (4 °C, 14,000× *g*, 15 min) were purified by affinity chromatography by mixing with 1 mL Ni-NTA agarose beads (FUJIFILM Wako), which were equilibrated with Lysis buffer in polyprep open columns (BIO-RAD, Hercules, CA, USA). After washing with Lysis buffer, proteins were eluted in elution buffer (50 mM Tris-HCl, pH 7.6, 500 mM NaCl, 400 mM imidazole). The elution fractions were mixed with 80% glycerol to reach the final 10% glycerol, and then homemade Ulp1 was added to the purified proteins (1/100 *w*/*w*) and incubated at 4 °C for 16 h. After the Ulp1 digestion, buffers of the fractions were replaced with anion exchange buffer (20 mM HEPES-KOH, pH 7.6, 180 mM GluK, 20 mM imidazole) by Amicon filter units (Amicon Ultra-10k, 30K, and 50K, Merck Millipore, Billerica, MA, USA) to decrease the concentration of imidazole and NaCl. The digested His-SUMO tag and Ulp1 were removed by 1 mL Ni-NTA agarose beads. The flowthrough fractions were purified by anion exchange chromatography using 1 mL Q sepharose high performance beads (Cytiva, Tokyo, Japan), which were equilibrated with 20 mM HEPES-KOH, pH 7.6. Proteins were eluted by stepwise elution with 1 CV of 20 mM HEPES-KOH, pH 7.6, with 0–1 M NaCl. The buffer of the eluted fractions was replaced with PURE storage buffer (20 mM HEPES-KOH pH 7.6, 180 mM GluK, 30% glycerol) by Amicon filter units. The concentrations of the glycolytic enzymes were quantified by a Bradford assay. Proteins were stored at −30 °C until use. 

### 4.3. Expression and Purification of Green Glifon4000 and MaLionR

The Green Glifon4000 gene with a 6xHis tag on its N-term, as an artificial gene synthesis product with codon optimization (Twist BioScience, South San Francisco, CA, USA), was cloned into the NdeI-XhoI site of the pET28a(+) vector. The *E. coli* BL21 (DE3) RIPL cell that carries the plasmid was cultivated at 20 °C for 4 days in LB medium. Because leaky expression without IPTG in the case of the medium consisting of bactotryptone from Nacalai Tesque was at a high level, no IPTG was added for Green Glifon4000 expression. After cell collection, Green Glifon4000 was purified by Ni-NTA agarose beads in the same manner as the glycolytic genes. For MaLionR purification, *E. coli* BL21 (DE3) RIPL cells harboring a plasmid encoding MaLionR [27] were cultivated at 20 °C for four days in LB medium. After cell collection, MaLionR was purified by Ni-NTA agarose beads and Q sepharose high-performance beads in the same manner as the glycolytic genes. Buffers of the purified proteins were replaced by a PURE storage buffer. Protein concentrations were quantified by CBB-stained SDS-PAGE gel using BSA as a standard. Proteins were stored at −30 °C until use.

### 4.4. In Vitro Reconstitution of Glycolysis

For the measurement of glucose and ATP concentrations, a reaction mixture that consisted of custom Sol1 of PURE*frex* 2.0 (-CP, -NTPs, -Mg(OAc) _2_); 16 mM Mg(OAc)_2_; 1.3 mM ATP; 1 mM NAD^+^; 60 mM KP_i_; 0.1 mM TPP; 50 mM glucose; various concentrations of Glk; 1 µM Pgi, FbaA, TpiA, GAPDH, Pgk, GpmA, Eno, and PDC; 2.5 µM PfkA, PykF, and PykA; 10 µM ADH; 2 µM Green Glifon4000; 4 µM MaLionR; and 50 mM glucose was incubated at 30 °C in qTOWER^3^G (Analytic Jena, Jena, Germany). Fluorescence intensities of Green Glifon4000 and MaLionR were periodically detected by qTOWER^3^G. Glucose was added just before initiating the reaction.

For the investigation of the effect of glycolytic intermediates, 0.1 µM Glk and 50 mM KP_i_ were used. For the quantification of ethanol synthesized by the reaction, Green Glifon4000 and MaLionR were omitted from the mixture above, and the reaction temperature was 37 °C, the same as the condition for SG-PURE 2.0.

### 4.5. G-PURE and SG-PURE Reactions

For the investigation of the P_i_ dependence of G-PURE 1.0, 2.0, SG-PURE 1.0, and 2.0, a reaction mixture that consisted of custom PURE*frex* 1.0 or 2.0 (-CK, -CP, -NTPs, -Mg(OAc)_2_); 15 mM Mg(OAc)_2_; 1.3 mM ATP; 1 mM GTP; 0.625 mM CTP; 0.625 mM UTP; 1 mM NAD^+^; various concentrations of KP_i_; 0.1 mM TPP; 1 µM Glk, Pgi, FbaA, TpiA, GAPDH, Pgk, Eno, and PDC; 2.5 µM PfkA, PykF, and PykA; 10 µM ADH; and 25 mM G6P or glucose was incubated at 37 °C in qTOWER^3^G. As the template DNA, the PCR product of sfGFP or SUMO-msfGFP fusion proteins [46] created by amplifying the region between the T7 promoter and T7 terminator was used at 2.8 nM. The templated DNA was purified with a PCR purification kit (Mini Plus Coulmn set, Viogen, New Taipei City, Taiwan).

For SG-PURE 2.0, reactions were conducted under the same conditions as for G-PURE, other than the fact that 0.1 µM Glk, 16 mM Mg(OAc)_2_, 50 mM KP_i_, and 50 mM glucose were used if not indicated otherwise. Total GFP fluorescence during reactions was detected in qTOWER^3^G. The fluorescence intensities of the band of proteins with GFP (non-boiling samples after the SG-PURE reaction) were detected by ChemiDoc Touch MP (BIORAD, Hercules, CA, USA). Protein expression was also verified by SDS-PAGE using a boiled sample with CBB staining.

### 4.6. Estimation of Glucose Depletion Time and ATP Recovery Time of Glycolysis

For the estimation of the glucose depletion time, the minimum slopes (*S_min_*) of glucose concentration dynamics were calculated. For the calculation, the time and y values that give *S_min_* were named *t*_1_ and *y*_1_. Glucose depletion time was defined as the time at which the line with slope *S_min_* passing through (*t*_1_, *y*_1_) and the minimum y value of the glucose concentration dynamics (y_2_) intersect. For the estimation of ATP recovery time, the maximum slopes (*S_max_*) of the ATP concentration dynamics were calculated. The time and y values that give *S_max_* were named *t*_1_ and *y*_1_. ATP recovery time was defined as the time at which the line with slope *S_max_* passing through (*t*_1_, *y*_1_) and the minimum y value of the ATP concentration dynamics (*y*_2_) intersect. A detailed illustration is given in Figure S6.

### 4.7. Quantification of Ethanol

Ethanol was quantified through the enzymatic method we used with slight modifications [47]. Briefly, a 2× quantification mixture (200 mM Tris-HCl, pH 7.6, 75 mM phenol, 15 mM 4-aminoantipyrine, 0.04 mg/mL alcohol oxidase, and 0.04 mg/mL peroxidase) was mixed with the reaction mixture of glycolysis or SG-PURE 2.0 and incubated at 37 °C in Biometra TRIO (Analytic Jena). After the incubation, the solution was moved to a 96-well microplate, and the absorbance at 510 nm of each sample was measured with a GloMax microplate reader (Promega, Madison, WI, USA).

## Data Availability

The data and materials in this study are available from the corresponding author upon reasonable request.

## Supplementary Materials

The following supporting information can be downloaded at https://www.mdpi.com/article/10.3390/molecules29132956/s1: Figure S1: Glycolysis pathway reconstituted in this study; Figure S2: Standard curves of fluorescent sensor proteins used in this study; Figure S3: Full image of Figure 3D; Figure S4: Ethanol yields of glycolysis and SG-PURE 2.0; Figure S5: Various protein expressions by PUREfrex 2.0 and their SDS-PAGE results; Figure S6: Estimation of glucose depletion time and ATP recovery time.
