# Supplementary material for: Cell-Free Protein Expression by a Reconstituted Transcription–Translation System Energized by Sugar Catabolism
Source: Molecules. 2024 Jun 21;29(13):2956. doi: 10.3390/molecules29132956 (PMC11243612; doi:10.3390/molecules29132956)
Supplement: Supplementary file 1 [file molecules-29-02956-s001.zip › molecules-3009972-supplementary.pdf]

Supplementary Information

Cell-free protein expression by a reconstituted transcription-translation system energized by sugar catabolism

Gaku Sato, Shintaro Miyazawa, Nobuhide Doi, Kei Fujiwara

Department of Biosciences & Informatics, Keio University, 3-14-1 Hiyoshi, Kohoku-ku, Yokohama 223–8522, Japan.

* Corresponding author
Kei Fujiwara: fujiwara@bio.keio.ac.jp

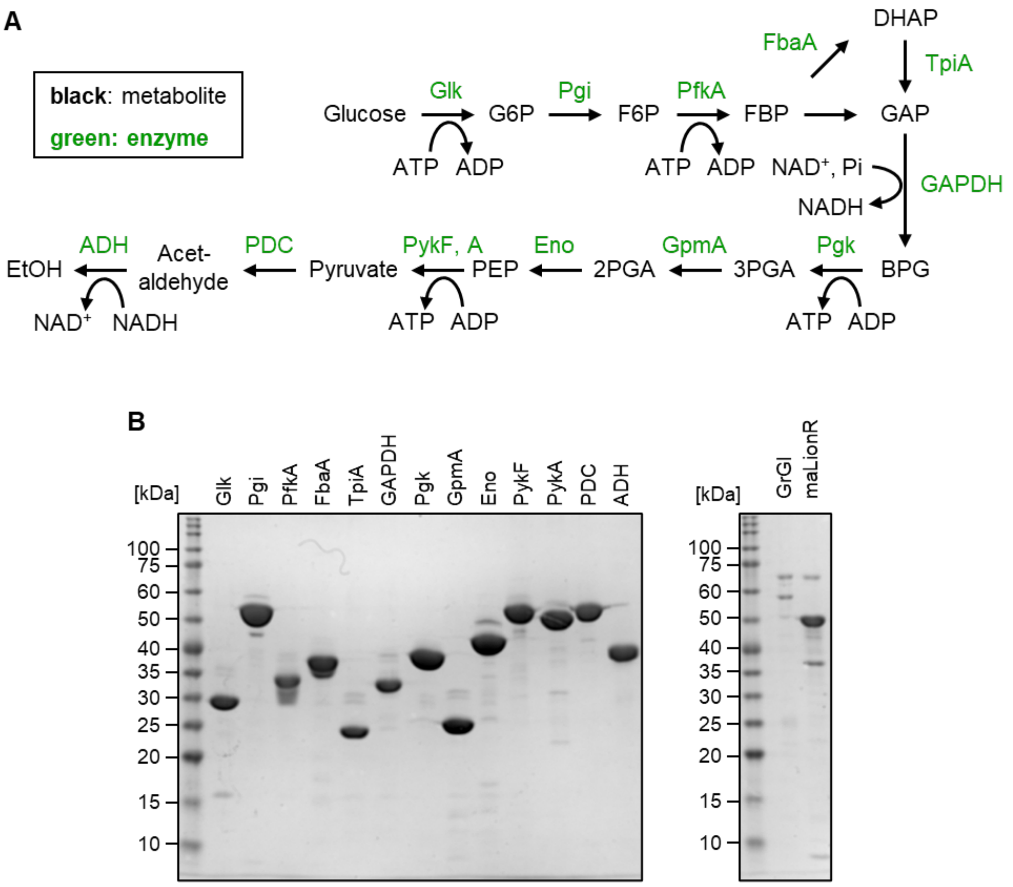

Fig. S1. Glycolysis pathway reconstituted in this study. (A) Detailed schematics of glycolysis. (B) SDS-PAGE image of purified proteins used in this study. GrGl is the abbreviation for Green Glifon4000.

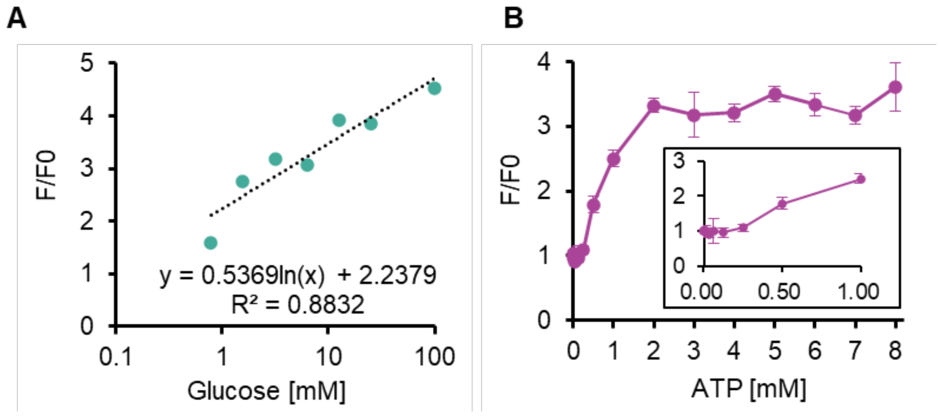

Fig. S2. Standard curves of fluorescent sensor proteins used in this study. (A) A standard curve of Green Glifon4000 at 30°C. F0 is the fluorescence of 0 mM Glucose (n=1). (B) A standard curve of MaLionR at 30°C. F0 is the fluorescence of 0 mM ATP. The inset shows F/F0 of 0.01-1 mM ATP. Means and standard deviations are shown (n=3).

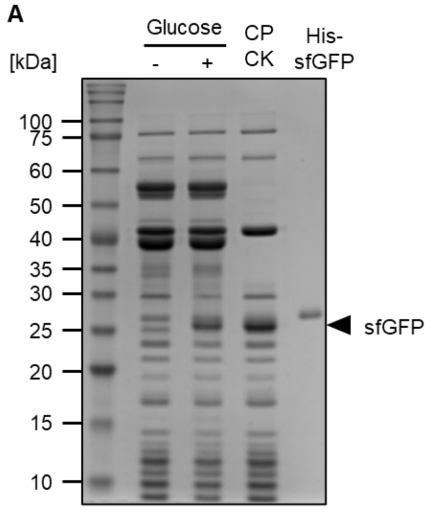

Fig. S3. Full image of Fig. 3D. (A) Boiled SDS-PAGE and CBB staining. (B) Non-Boiled SDS-PAGE.

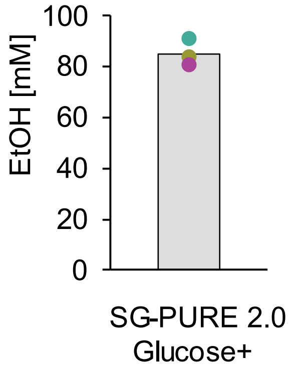

Fig. S4. Ethanol yields SG-PURE 2.0. The bars represent the average, and the dots represent the individual values of triplicates. The theoretical maximum yield of ethanol synthesized from 50 mM glucose is 100 mM.

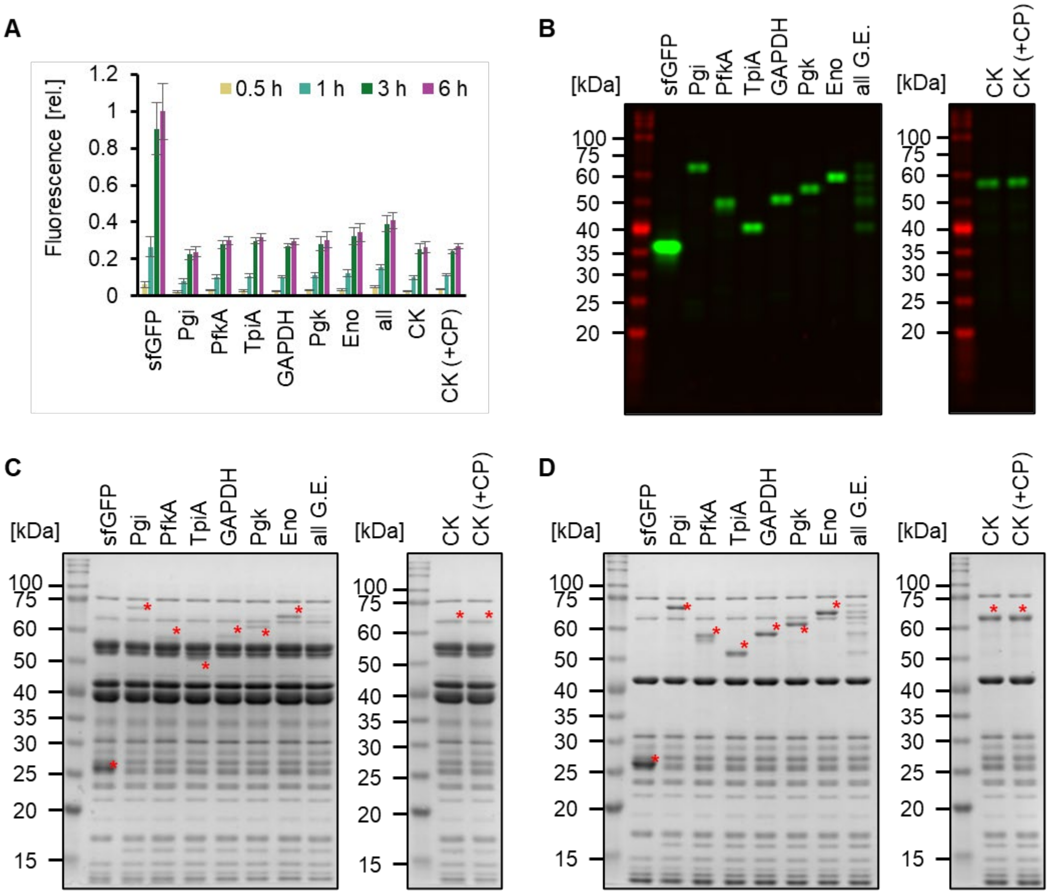

Fig. S5. Various protein expression by PUREfrex 2.0 and their SDS-PAGE results. (A) Time course of synthesis levels of various sfGFP-tagged proteins by PUREfrex 2.0. Fluorescence of proteins were detected by qTower3G. Means and standard deviations are shown (n=3). (B) Non-boil SDS-PAGE of various proteins expressed by PUREfrex 2.0. (C) SDS-PAGE of various proteins expressed by SG-PURE 2.0. (D) SDS-PAGE of various proteins expressed by PUREfrex 2.0.

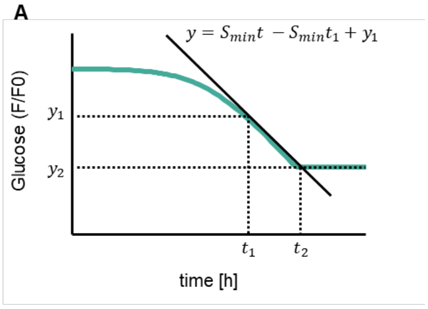

$$Glucose\ depletion\ time = t_2 = t_1 + \frac{y_2 - y_1}{S_{min}}$$

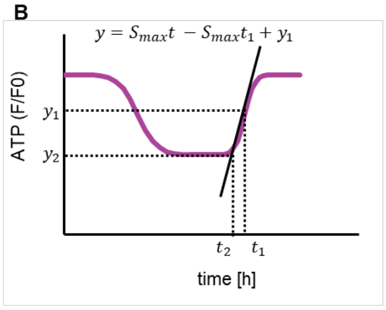

$$ATP\ recovery\ time = t_2 = t_1 - \frac{y_1 - y_2}{S_{max}}$$

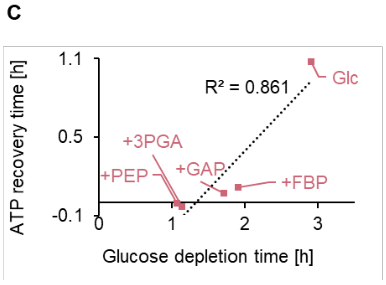

Fig. S6. Estimation of glucose depletion time and ATP recovery time. (A) Illustration of glucose depletion time estimation. (B) Illustration of ATP recovery time estimation. Details are described in Methods. (C) Scattered plot of ATP recovery time against glucose depletion time.
